# Supplementary material for: Pannexin-1 Modulates Inhibitory Transmission and Hippocampal Synaptic Plasticity
Source: Biomolecules. 2023 May 25;13(6):887. doi: 10.3390/biom13060887 (PMC10296222; doi:10.3390/biom13060887)
Supplement: Supplementary file 1 [file biomolecules-13-00887-s001.zip › biomolecules-2408310-supplementary.pdf]

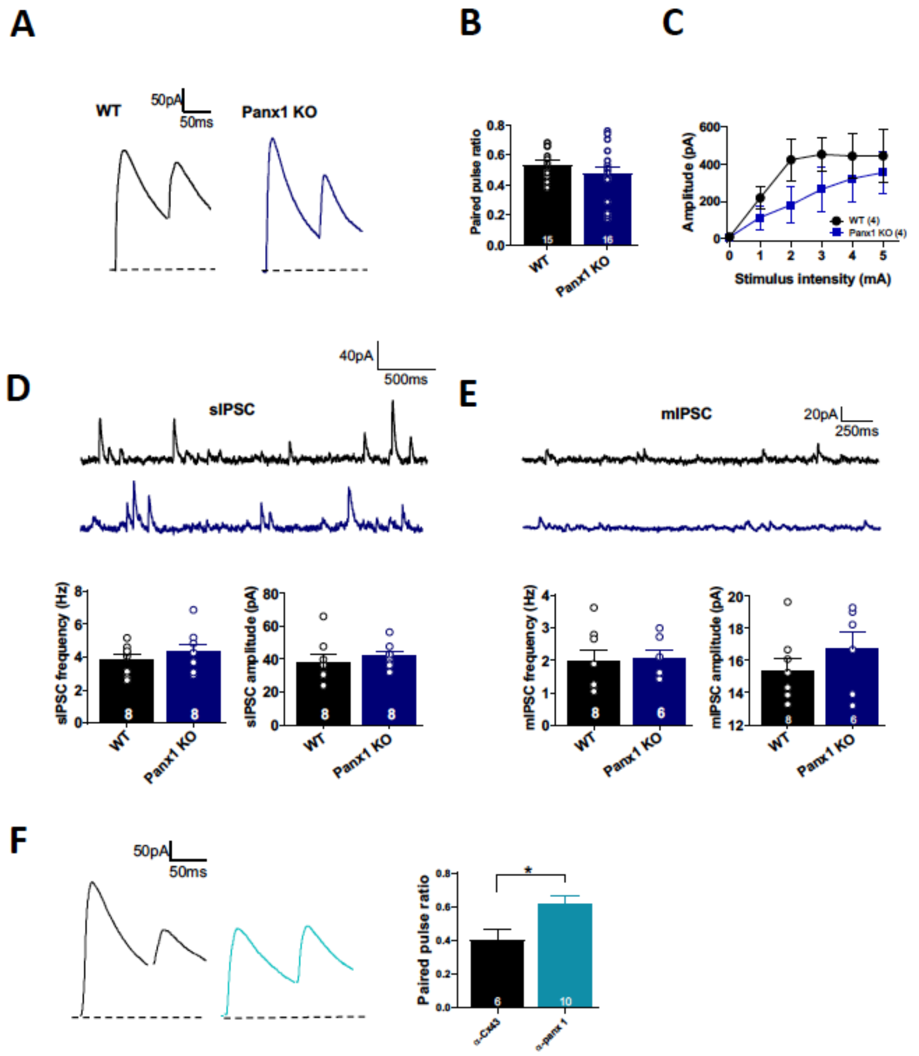

Figure S1. Absence of Panx1 did not change basal GABAergic transmission: (**A**) Samples traces from evoked IPSC and PPR for pyramidal neurons recorded from wt mice (black) and Panx1 KO mice (blue). PPR (**B**) and IPSCs amplitude (**C**) were no different between groups. (**D**) Quantitative analyses of sIPSC frequency (**left**) and amplitude (**right**) did not show significant differences be-tween control and Panx1 KO mice. (**E**) Absence of Panx1 did not affect either mIPSC frequency or mIPSC amplitude compared with WT mice. (**F**) Representative traces of PPR by using α-Cx43 (black) and α-panx1 (skyblue) in the patch pipette. PPR values were significantly higher in the presence of α-panx1 compared with control condition (α-Cx43). Data are performed as mean ± SEM. Number of recorded cells is indicated within bars.

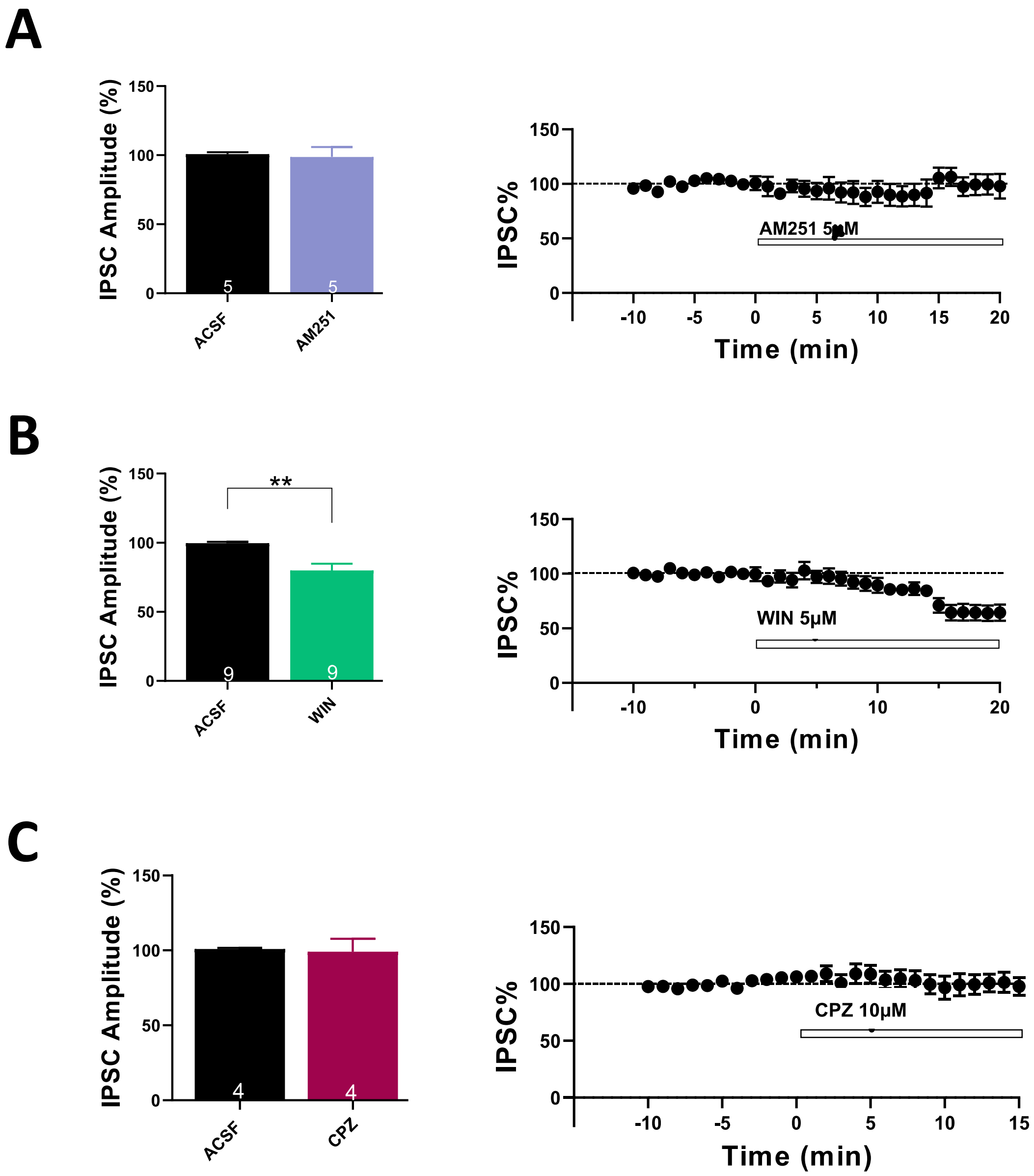

Figure S2. Effect the eCB on basal GABA transmission. (**A**) AM251 perfusion has no effect on basal GABAergic synaptic transmission. (**B**) Effect of WIN 55,212-2 on evoked IPSCs in a CA1 pyramidal neuron. (**C**) the specific TRPV1 antago-nist capsazepine (CPZ, 10 μM), bath application of 10Panx1 did not affect IPSC amplitude.
